# Transjejunal ERCP through an endoscopic ultrasound-guided jejuno-duodenal anastomosis in a patient with gastric bypass without excluded stomach

**DOI:** 10.1055/a-2155-4622

**Published:** 2023-09-15

**Authors:** Nada El-Domiaty, Hadrien Alric, Alessandro Di Gaeta, Elena Tenorio-González, Christophe Cellier, Gabriel Rahmi, Enrique Pérez-Cuadrado-Robles

**Affiliations:** 1 Department of Gastroenterology, Georges-Pompidou European Hospital, AP-HP Centre, Paris, France; 2 Endemic Medicine Department, Faculty of Medicine, Helwan University, Cairo, Egypt; 3 University of Paris-Cité, Paris, France; 4 Department of Interventional Radiology, Georges-Pompidou European Hospital, AP-HP Centre, Paris, France

Gallbladder stones after bariatric surgery are not uncommon. Roux-en-Y gastric bypass (RYGB) renders standard endoscopic retrograde cholangiopancreatography (ERCP) unfeasible. Endoscopic ultrasound (EUS)-directed transgastric ERCP (EDGE) using a lumen-apposing metal stent (LAMS) is gaining ground in this setting 1 2 and has been proposed in recent European guidelines 3 . In addition, more is known about how to perform EDGE in patients with a standard RYGB anatomy 4 , than in patients with non-RYGB 5 or atypical gastric bypass such as those without remnant stomach. This case shows a modified EDGE procedure in this latter scenario.

A 79-year-old woman presented with severe acute biliary cholangitis suspected to be due to ampulloma with biliary stones. She had a history of sleeve gastrectomy, which was converted to RYGB with further surgical gastrectomy of the excluded stomach. EUS was first used to attempt a one-shot EDGE procedure; however, it was difficult to locate the excluded duodenum and the one-shot procedure through a jejuno-duodenal anastomosis was considered risky. Thus, percutaneous transhepatic biliary drainage (PTBD) was planned.

At 48 hours, EUS-guided jejuno-duodenostomy was performed, assisted by PTBD connected to a water pump ( Fig. 1 ). A freehand EUS-guided anastomosis was created using a 20 mm LAMS (Hot Axios; Boston Scientific, Marlborough, Massachusetts, USA), similarly to the wireless EUS-guided gastroenterostomy simplified technique 1 ( Video 1 ). Upper gastrointestinal endoscopy through the EUS-guided anastomosis was carefully performed to confirm the passage. Interestingly, transjejunal diagnostic EUS in an inverted anatomy from D3 to D4 was performed using the transpapillary drainage tube to guide the examination, excluding an ampulloma and revealing multiple biliary stones. ERCP combined with biliary sphincterotomy using an inverted sphincterotome allowed successful stone extraction ( Fig. 2 ). The percutaneous drainage tube was removed. The patient recovered well with no recurrent cholangitis or adverse events over a follow-up of 3 months.

**Fig. 1:**
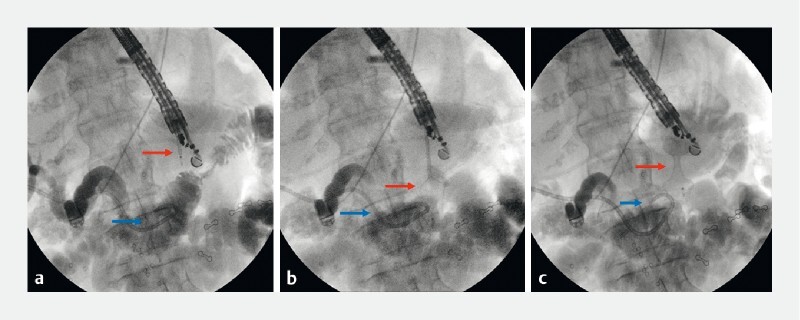
Endoscopic ultrasound (EUS)-guided jejuno-duodenal anastomosis was guided by a percutaneous transhepatic biliary drainage tube (blue arrows) connected to a water pump. A freehand EUS-guided anastomosis using a 20 mm lumen-apposing stent (red arrows) was performed under fluoroscopy control.

**Fig. 2:**
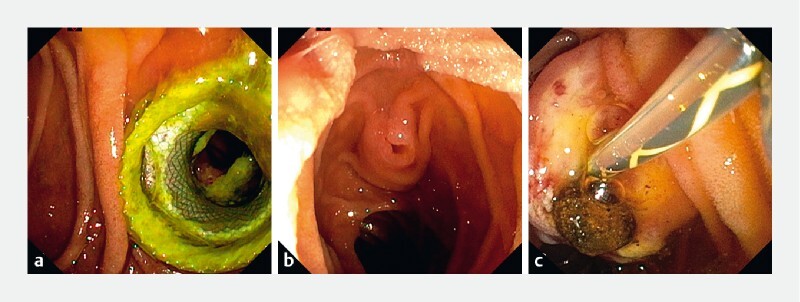
Endoscopic views. **a** The endoscopic ultrasound-guided anastomosis. **b** Transjejunal endoscopic retrograde cholangiopancreatography showing an inverted papilla. **c** An inverted sphincterotome combined with further stone extraction.

**Video 1** Endoscopic ultrasound-guided jejuno-duodenal anastomosis assisted by percutaneous transhepatic biliary drainage connected to a water-pump and further transjejunal endoscopic retrograde cholangiopancreatography in a patient with an atypical gastric bypass presenting with acute cholangitis.


Endoscopy_UCTN_Code_TTT_1AS_2AD

## Notes

Endoscopy E-Videos

https://eref.thieme.de/e-videos
*E-Videos* is an open access online section of the journal *Endoscopy* , reporting on interesting cases and new techniques in gastroenterological endoscopy. All papers include a high-quality video and are published with a Creative Commons CC-BY license. Endoscopy E-Videos qualify for HINARI discounts and waivers and eligibility is automatically checked during the submission process. We grant 100% waivers to articles whose corresponding authors are based in Group A countries and 50% waivers to those who are based in Group B countries as classified by Research4Life (see: https://www.research4life.org/access/eligibility/ ). This section has its own submission website at https://mc.manuscriptcentral.com/e-videos
